# Nano-Based Technology in Glioblastoma

**DOI:** 10.3390/molecules30173485

**Published:** 2025-08-25

**Authors:** Dorota Bartusik-Aebisher, Izabela Rudy, Karolina Pięta, David Aebisher

**Affiliations:** 1 Department of Biochemistry and General Chemistry, Faculty of Medicine, Collegium Medicum, University of Rzeszów, 35-959 Rzeszów, Poland; dbartusikaebisher@ur.edu.pl; 2 Student Scientific Club of Biochemists URCell, Faculty of Medicine, Collegium Medicum, University of Rzeszów, 35-959 Rzeszów, Poland; akacja03@interia.pl (I.R.); karolinapietax@gmail.com (K.P.); 3 Department of Photomedicine and Physical Chemistry, Faculty of Medicine, Collegium Medicum, University of Rzeszów, 35-959 Rzeszów, Poland

**Keywords:** central nervous system, glioblastoma, nanotechnology

## Abstract

Neoplasms of the central nervous system (CNS) constitute a minor fraction of all malignant tumors. CNS accounts for approximately 4% of newly diagnosed oncological cases. Among primary CNS neoplasms, gliomas predominate, comprising nearly 90% of all malignant brain tumors, with Glioblastoma (GBM) representing the most prevalent and aggressive histological subtype. The earliest documented occurrences of GBM date back to the 19th century. Contemporary therapeutic modalities for GBM primarily involve maximal surgical resection, adjuvant radiotherapy, and systemic chemotherapy. However, the intrinsic heterogeneity of GBM poses a formidable obstacle to treatment efficacy. The immunosuppressive tumor microenvironment, coupled with the restrictive nature of the blood–brain barrier (BBB), significantly limits the intratumorally delivery of chemotherapeutic agents. The emergence of nanotechnology in the biomedical domain has been driven by the urgent need to develop more effective and targeted anticancer interventions. Optimizing therapeutic outcomes necessitates the concurrent application of multimodal strategies. This review emphasizes the Nano-Based Technology in GBM.

## 1. Introduction to Glioblastoma

**Figure 1:**
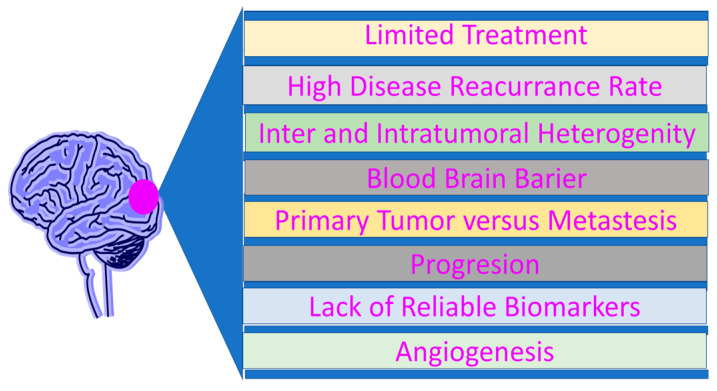
GBM characteristics.

Gliomas predominate among central nervous system tumors. They account for approximately 90% of all malignant brain tumors. Glioblastoma GBM is the most common primary malignant brain tumor [1]. It accounts for nearly 15% of all primary central nervous system tumors and approximately 50% of all malignant primary central nervous system tumors [2]. According to the World Health Organization (WHO) classification, GBM is classified as a grade IV malignancy [1]. It is characterized by a high proliferation rate and significant invasive potential, contributing to a poor prognosis despite aggressive therapeutic interventions [3]. Median overall survival is approximately 15 months from diagnosis [1]. GBM most commonly occurs in the brain, primarily in the frontal and temporal lobes, and to a lesser extent in the occipital and parietal lobes. However, it can also occur in the brainstem, cerebellum, or spinal cord [4]. Although GBM can occur at any age, it is more frequently diagnosed in older adults. Its incidence increases with age, peaking between ages 75 and 84, with a median age of onset of 64 [1]. Among them, rare exposure to ionizing radiation—particularly as part of pediatric radiotherapy for malignancies—is noted. There is also a genetic predisposition to GBM development in patients with Lynch syndrome, Turcot syndrome type 1, and Li-Fraumeni syndrome [5]. Epidemiological data indicate a higher incidence in men compared to women and among Caucasian individuals, in contrast to other racial and ethnic populations. No associations have been confirmed between GBM incidence and lifestyle-related risk factors such as dietary habits or tobacco use [6]. Some studies have suggested a possible link between viral infections—such as simian virus 40 (SV40), human herpesvirus 6 (HHV-6), and cytomegalovirus (CMV)—and GBM pathogenesis. However, this hypothesis remains unproven and is considered controversial by many researchers [7]. The clinical presentation of GBM is largely nonspecific. Patients most commonly report headaches, nausea, and behavioral changes [8]. Nausea often occurs due to the so-called “mass effect,” which is caused by increased intracranial pressure (ICP) [9]. Infection may resemble ischemic stroke, and characteristic symptoms are a consequence of neurological complications, cognitive impairment, and urinary incontinence [10]. Neurological treatment depends on the tumor’s location. Impaired information and failure to respond to external stimuli may result from frontal lobe failure, while tumors detached in the cerebral hemisphere may cause speech disorders [9]. Rapid device activity—revealing dysphagia and warning signs—often occurs about a week before death [8]. Histopathological confirmation by tumor biopsy is required based on the severity of the lesion, histological architecture, and molecular profile [7]. Advanced MRI methods, such as diffusion-weighted imaging (DWI) and perfusion imaging, enable assessment of tumor cellularity and vascularity. GBM is characterized by hypercellularity and high vascularity due to its aggressive nature [11]. As an additional diagnostic tool, positron emission tomography (PET)—especially with the use of characteristic markers—offers distinction but is not metabolically marked for tumor activity [6]. PET with 18F-fluorodeoxyglucose (FDG) has certain limitations due to the high physiological uptake of FDG in normal brain tissue, which complicates the evaluation of tumor metabolism. Therefore, amino acid–based tracers such as 18F-fluoroethyl-L-tyrosine (FET), 11C-methionine (MET), and 18F-DOPA are currently preferred, as they are selectively taken up by neoplastic cells and enable better delineation of glioma tissue from surrounding healthy neurons [12]. Cerebrospinal fluid (CSF) analysis, obtained via lumbar puncture, is another diagnostic modality applied in GBM. This form of liquid biopsy is viable due to the presence of tumor-derived cells in both the bloodstream and CSF, providing a less invasive alternative to tissue biopsy [7]. The WHO 2021 classification highlights the importance of molecular diagnostics in GBM. For instance, the identification of homozygous CDKN2A/B deletion is sufficient to assign a tumor to WHO grade IV, irrespective of the presence or absence of microvascular proliferation or necrosis [3]. Current treatment modalities include surgical resection, radiotherapy, and chemotherapy. Given the tumor’s invasive behavior and the presence of the blood–brain barrier (BBB), effective GBM treatment remains a formidable clinical challenge. Additional difficulties stem from the high degree of tumor heterogeneity and the frequent occurrence of recurrence. The adverse effects of radiotherapy further complicate management and are categorized based on time of onset into acute, early delayed, and late delayed toxicities [13]. Surgical resection is particularly challenging due to the frequent localization of GBMs near eloquent brain regions governing speech, motor functions, and sensory processing [9]. The Stupp protocol remains the standard-of-care for patients with newly diagnosed GBM. It involves maximal safe resection, followed by concurrent radiotherapy and daily temozolomide (TMZ) at 75 mg/m^2^ for six weeks, along with six cycles of maintenance TMZ (150–200 mg/m^2^ for five days per month) [13]. Second-line chemotherapy is individualized based on the patient’s clinical status and tumor profile [14]. Antiangiogenic therapy with bevacizumab—a monoclonal antibody targeting circulating vascular endothelial growth factor A (VEGF-A) and inhibiting its interaction with VEGF receptors (VEGFR)—is reserved for recurrent GBM cases [15]. Other agents with demonstrated therapeutic activity include lomustine, carmustine, and the PCV regimen (procarbazine, lomustine, vincristine). Despite aggressive treatment, GBM remains a highly refractory malignancy, with recurrence occurring in 75–90% of cases. Further innovation and refinement of treatment strategies are essential to improve survival outcomes and quality of life for patients [6]. The summary of GBM factors is presented below (Figure 1).

### 1.1. Overview of Nanotechnology

Conventional anticancer therapies are associated with numerous limitations; consequently, there is growing interest in the application of nanotechnology in medicine—one of the most promising technological advancements of the 21st century [16]. Advances in the understanding of cancer biology are increasingly directing research efforts toward the use of nanoparticles as a novel therapeutic and diagnostic strategy. One of the current challenges in oncology is the effective delivery of anticancer agents to each of these components [17]. In the case of GBM, an additional obstacle is the presence of the blood–brain barrier (BBB), which prevents the passage of nearly all small- and large-molecule drugs. The application of nanotechnology in GBM therapy enables the circumvention of barriers such as prolonged systemic circulation, trans-BBB transport, and controlled intracellular drug release within tumor cells [18]. Depending on their degree of spatial confinement, nanomaterials are classified into four types: zero-dimensional nanomaterials, in which all dimensions are on the nanometer scale (e.g., nanoparticles); one-dimensional nanomaterials, in which any one of the three dimensions is at the nanoscale (e.g., nanotubes, nanowires); two-dimensional nanomaterials, in which any two of the three dimensions are on the nanometer scale (e.g., nanosheets, nanoplates, and nanocoatings); and three-dimensional nanomaterials, in which all dimensions are within the nanoscale range, allowing electrons to move freely without restriction in any direction. Other classification systems are based on origin, porosity, phase, and dispersion properties [19]. Nevertheless, chemical composition remains the most widely used criterion for categorizing nanomaterials. Based on this, they are typically classified as organic, inorganic, carbon-based, or composite materials [20]. Nanomedicine is primarily applied in the efficient delivery of anticancer drugs, as well as in diagnostics and imaging [21].

### 1.2. Aim of the Review

The aim of this review is to analyze the potential application of nanotechnology in the treatment of GBM: exploring novel diagnostic and therapeutic perspectives and enhancing the overall efficacy of treatment strategies (Figure 1). This review presents key information regarding the molecular biology of GBM, including genetic mutations, signaling pathways, and tumor microenvironmental characteristics. It outlines the major barriers that hinder effective therapeutic intervention. Both standard treatment modalities—such as surgical resection, chemotherapy, and radiotherapy—and innovative approaches are discussed, with particular emphasis on nanotechnology-based solutions. The mechanisms of action of nanoparticles in GBM therapy, as well as the various types of nanomaterials employed for this purpose, are described. The study also presents examples of ongoing research and clinical trials. The primary objective is to assess how advanced nanotechnological approaches can improve current treatment methods for GBM by enhancing therapeutic precision and efficacy while minimizing adverse effects.

## 2. GBM: Characteristics and Therapeutic Challenges

### 2.1. Biology of GBM

#### 2.1.1. Pathophysiology of GBM: Neoplastic Cells, Angiogenesis, and Tumor Heterogeneity

**Figure 2:**
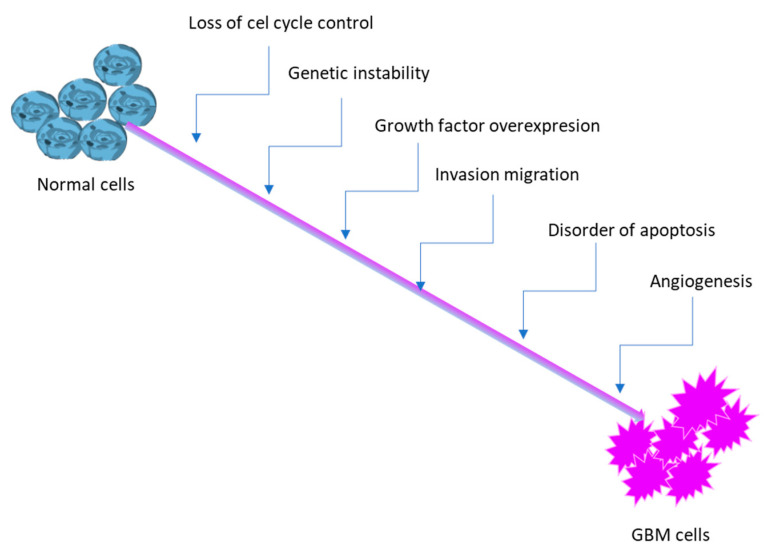
The pathway of GBM signaling.

Angiogenesis is the process of forming new blood vessels from existing ones, crucial for tumor growth and metastasis. In gliomas, particularly GBM, angiogenesis is a hallmark, driven by factors like hypoxia and leading to the formation of a complex, heterogeneous tumor microenvironment (TME). This TME includes various cell types and niches with distinct characteristics, contributing to the tumor’s aggressive nature and resistance to therapy [22]. In cancer, angiogenesis becomes dysregulated, leading to excessive and abnormal blood vessel formation. GBM, is a highly vascularized tumors, relying heavily on angiogenesis for growth and progression. These niches contain diverse cell populations, including cancer cells, endothelial cells, immune cells (like microglia and macrophages), and other cell types. Several factors drive glioma angiogenesis, including hypoxia-inducible factor 1 (HIF-1α), vascular endothelial growth factor (VEGF), fibroblast growth factor (FGF), and angiopoietins. The profile of the pathway of GBM is presented below (Figure 2).

The first recognized cases of GBM were reported in the late 19th century. At the time, neither the cellular origin nor the structure of tumor tissue was understood; however, the infiltrative nature of the tumor within healthy brain parenchyma had already been described. In 1865, Rudolf Virchow introduced the term “glioma” into medical nomenclature based on light microscopy findings [23]. In 1930, German neurologist Hallervorden proposed a potential association between multiple sclerosis and GBM multiforme. Hugo Ribbert, in an attempt to elucidate the origin of gliomas, formulated a hypothesis grounded in embryonic neurogenesis and the role of embryonic stem cells. Bailey and Cushing developed a classification of gliomas based on the degree of cellular anaplasia, asserting that tumors previously classified as spongioblastoma multiforme were not of glial origin. The connection between astrocytic tumors and GBM was later demonstrated by Scherer, leading to the formal definition of GBM multiforme as a malignancy arising from pre-existing astrocytomas. In contrast, tumors lacking a lower-grade astrocytic precursor were classified as primary GBMs. Currently, the distinction between primary and secondary GBMs hinges primarily on the mutational status of the isocitrate dehydrogenase (IDH) gene, which is typically altered in secondary GBMs [3,23]. Primary GBMs are significantly more prevalent, comprising approximately 90% of all GBMs. They predominantly affect older adults. The remaining cases—secondary GBMs—arise from the malignant progression of lower-grade astrocytomas and are more frequently observed in younger patients. Notably, the adult brain retains populations of cells with proliferative potential, suggesting that GBM may originate from neural stem cells [23]. This hypothesis was substantiated experimentally: Lee et al. demonstrated that oncogenic stimulation of fetal neural stem cells via H-ras resulted in neoplastic transformation. In contrast, oligodendrocytes derived from v-myc–expressing neural stem cells did not undergo malignant transformation following the same oncogenic stimulus [24]. Key genetic alterations in GBM include loss of heterozygosity (LOH) on chromosome 10q, observed in 60–90% of cases, and deletions affecting the p53 gene, which are present in approximately 85–87% of tumors [25,26,27].

#### 2.1.2. IDH Mutation

IDH mutations are present in nearly all cases of secondary GBM, whereas they occur infrequently in primary gliomas [3]. Isocitrate dehydrogenase (IDH) is an enzyme that plays a pivotal role in regulating the tricarboxylic acid (TCA) cycle. Three isoforms of IDH are recognized: IDH1, which is localized in the cytoplasm and peroxisomes; and IDH2 and IDH3, which are found in the mitochondrial matrix [28].

Isocitrate dehydrogenase catalyzes the chemical reactions:

Isocitrate + NAD^+^ ⇌ 2-oxoglutarate + CO_2_ + NADH + H^+^

Isocitrate + NADP^+^ ⇌ 2-oxoglutarate + CO_2_ + NADPH + H^+^

The overall free energy for this reaction is −8.4 kJ/mol.

IDH1 and IDH2 catalyze the oxidative decarboxylation of isocitrate and NADP^+^ to produce α-ketoglutarate (α-KG), NADPH, and carbon dioxide. This multi-step process involves the oxidation of isocitrate to oxalosuccinate, followed by its decarboxylation to α-KG, which serves as a crucial cofactor for numerous cellular enzymes [29]. The IDH gene mutation affects a critical arginine residue that is essential for isocitrate recognition [30]. Specifically, the positively charged arginine at position 132 is substituted with less polar amino acids, including histidine, lysine, or cysteine. This substitution impairs the formation of hydrogen bonds with the α- and β-carboxyl groups of isocitrate [31,32]. As a result, the enzyme loses its physiological function, and instead of converting isocitrate to α-KG, it produces 2-hydroxyglutarate (2-HG), while simultaneously exhibiting increased binding affinity for NADPH. These alterations lead to intracellular accumulation of 2-HG, a metabolite implicated in the initiation of oncogenesis [33]. Cellular concentrations of 2-HG have been shown to reach as high as 5–30 mM [34]. IDH1-mutant cells exhibit enhanced oxidative metabolism via the Krebs cycle and reduced reductive glutamine metabolism [35]. Additionally, glioma cells harboring IDH1 mutations demonstrate increased sensitivity to glutaminase inhibition, suggesting that glutaminolysis serves as a compensatory mechanism essential for maintaining metabolic homeostasis [36]. Moreover, IDH mutations are associated with diminished glycolytic activity and reduced glucose metabolism, leading to a slower tumor growth rate in IDH-mutant neoplasms [37]. In summary, IDH mutations result in profound metabolic reprogramming, which may provide a foundation for the development of targeted therapies aimed at exploiting these unique metabolic vulnerabilities in gliomas and other IDH-mutant cancers. GBM, mutations in the isocitrate dehydrogenase (IDH) gene are linked to a heightened sensitivity to ferroptosis, a form of cell death characterized by iron-dependent lipid peroxidation. Specifically, IDH mutations, particularly IDH1R132H, promote ferroptosis by increasing lipid peroxidation and reducing levels of GPX4, a key enzyme that protects against ferroptosis. This makes IDH-mutated GBM cells more vulnerable to ferroptosis-inducing agents [38,39]

#### 2.1.3. The Notch Signaling Pathway

Cellular processes such as differentiation, proliferation, and apoptosis of neural cells are tightly regulated by several signaling cascades, including the Notch pathway. This pathway plays a pivotal role in the differentiation of neural stem cells into mature glial cells [40]. Neural stem cells (NSCs) serve as the foundation for neurogenesis; genetic and functional alterations in these cells can contribute to the development of brain tumors [41]. The Notch signaling pathway comprises four receptors: Notch1, which may function as either a tumor suppressor or oncogene; Notch2, considered a prognostic marker in glioma; Notch3, which promotes glioma cell proliferation; and Notch4, associated with increased tumor aggressiveness [42]. Notch1 expression is significantly higher in patients with survival exceeding one year compared to those with shorter survival; however, excessive Notch1 expression has been correlated with poorer overall survival [3]. Each of these transmembrane receptors can interact with two major classes of ligands: Delta-like (Dll1–3 and –4) and Jagged (Jagged–1 and –2) [43]. During neurogenesis, Notch receptors are expressed on NSCs, while Delta ligands are transiently present on differentiating neurons. NSCs exposed to the Delta signal undergo Notch activation, promoting their differentiation into glial cells. Thus, activation of Notch receptors by Delta ligands plays an essential role in neurodevelopment [41]. This includes upregulation of growth-promoting metabolic regulators such as Myc [44]. Numerous genes have been shown to modulate the Notch pathway and influence the development and proliferation of GBM. For instance, CRMP5 inhibits receptor degradation, thereby sustaining Notch signaling and enhancing tumor growth [45]. In contrast, miR-34a inhibits GBM progression by downregulating Notch1 and Notch2 expression [46]. Suppression of RND3, a natural inhibitor of the Notch transcriptional complex, enhances Notch signaling and GBM cell proliferation [47]. Leptin is another factor implicated in tumor growth via activation of Notch1 effectors [48]. Elevated levels of NICD (Notch intracellular domain) have been observed in GBM patients, further promoting tumor cell proliferation [49]. LINC01152 activates MAML2 by recruiting SRSF1 and binding miR-466, thereby exacerbating GBM progression [50]. Notch signaling also plays a crucial role in GBM cell migration and invasion. Its influence on invasiveness is mediated through the transcriptional target gene Hes1 [51]. The downstream effector Hey1 suppresses USP11 expression, promoting tumor cell migration. Notch2-mediated activation of TNC expression further enhances GBM invasiveness via an RBPJK-dependent mechanism. A promising therapeutic strategy involves dual inhibition of the Notch pathway and CDK4 using resveratrol, which has been shown to reduce GBM invasiveness. Additionally, decreased levels of the transcription factor CBF1 result in attenuated Notch activity and diminished glioma cell invasiveness [44]. Lowering uPA/uPAR expression may inhibit the proteolytic cleavage of Notch receptors at the Gly1743-Val1744 site, thereby weakening downstream signaling. This ultimately suppresses NF-κB, ERK, and AKT signaling pathways and reduces GBM invasiveness [52]. Conversely, RBM8A enhances glioma invasiveness by upregulating Notch1 and STAT3 transcription, thereby activating the Notch/STAT signaling axis. Similarly, EIF4A3 supports GBM growth and invasion by modulating Notch1 expression through a STAT3-dependent mechanism [53].

#### 2.1.4. Platelet-Derived Growth Factor (PDGF)

Platelet-derived growth factor (PDGF) possesses a strong capacity to promote tumor cell proliferation and survival, making it an attractive therapeutic target in GBM [54]. In normal glial cells, PDGF signaling is initiated through the binding of ligands—such as PDGFA, PDGFB, and PDGFC—to specific receptors belonging to the tyrosine kinase receptor family: PDGFRα and PDGFRβ. The activated receptor complex then serves as a docking platform for multiple protein complexes, initiating a cascade of intracellular signaling pathways that ultimately lead to DNA synthesis and cellular proliferation [55,56]. In glioma cells, the existence of an autocrine PDGF signaling loop has been documented [57]. Tumor cells in GBM are characterized by PDGF overexpression, with PDGFA and PDGFB showing particularly high levels of expression, whereas PDGFC is the least expressed ligand in this context [58]. Studies conducted by Westermark revealed that the PDGFRα gene may undergo amplification, mutation, or chromosomal rearrangement in glioma tumors, all of which contribute to tumorigenesis [59]. Additionally, research by Shih et al. demonstrated that PDGF and PDGFR overexpression in glioma cell lines and tumor samples correlate with higher tumor grade and malignancy [55]. Further investigations by Popescu et al. indicated that both PDGF and its receptors regulate critical cellular processes in glioma, including proliferation, differentiation, and apoptosis [60]. Mangiola and colleagues conducted a study focused on the inhibition of PDGFRα and observed a significant reduction in spinal cord tumor cell proliferation by approximately 38 ± 9.5%, alongside a decrease in PDGFRα expression levels [61]. Collectively, these findings indicate that the PDGF signaling pathway is a well-characterized mechanism and represents a promising target for therapeutic intervention in GBM multiforme.

#### 2.1.5. Epidermal Growth Factor Receptor (EGFR)

The epidermal growth factor receptor (EGFR) is among the most frequently altered genes in GBM multiforme. Genetic aberrations affecting EGFR occur in over 50% of GBM cases, with a significantly higher prevalence observed in primary GBMs [62]. Amplifications and mutations of the EGFR gene, particularly within the extracellular domain, result in constitutive activation of downstream signaling pathways such as PI3K/AKT and MAPK, thereby promoting tumor cell proliferation, invasion, and angiogenesis [61]. One of the most common alterations is the EGFRvIII mutation, characterized by a deletion of exons 2 through 7. This leads to the production of a constitutively active receptor that functions independently of ligand binding. EGFRvIII is associated with poor prognosis and resistance to standard therapies. Other variants, such as EGFRvII and EGFRx, also contribute to the molecular heterogeneity of GBM and represent potential targets for therapeutic intervention [63].

#### 2.1.6. Ceramide Signaling

Sphingosine and free fatty acids are metabolic products of ceramides, generated through the enzymatic activity of acid ceramidase (ASAH1) [62]. It has been demonstrated that sphingolipids—such as sphingosine-1-phosphate (S1P), a direct metabolite—are key signaling mediators involved in regulating cell proliferation, whereas ceramides promote cellular senescence and apoptosis [64,65]. Ceramide (Cer), a central metabolite within the sphingolipid (SL) signaling network, functions as a tumor-suppressive lipid capable of eliciting antiproliferative and pro-apoptotic responses [65]. Histological studies have confirmed a metabolic shift in GBM (GBM) cells from ceramide toward S1P synthesis, resulting in elevated S1P levels at the expense of ceramide concentrations [66]. Additionally, a modified form of ASAH1 in GBM may be secreted into the extracellular space, thereby transmitting tumorigenic potential to neighboring cells [67]. Earlier studies have shown that ASAH1 is strongly expressed in glioma cells. Doan et al. observed that ASAH1 expression levels were higher in irradiated glioma cell cultures and tumor tissues compared to non-irradiated counterparts, correlating with increased resistance to apoptosis and tumor recurrence [66]. Although no clinically approved drugs currently exist that directly target ceramide signaling in gliomas, several ASAH1 inhibitors—such as carmofur, N-oleoylethanolamine, and ARN14988—have demonstrated efficacy in vitro across multiple glioma cell lines, including U87 and patient-derived cells. Notably, the antitumor activity of ASAH1 inhibitors has surpassed that of temozolomide (TMZ)—an FDA-approved chemotherapeutic—suggesting that pharmacological inhibition of ASAH1 may increase ceramide accumulation in tumor tissue and induce apoptosis in glioma cells [68,69].

### 2.2. Mechanisms of Drug Resistance in GBM, Including the Blood–Brain Barrier

The presence of the blood–brain barrier (BBB) and the blood–brain tumor barrier (BBTB) constitutes a significant obstacle in the chemotherapeutic treatment of brain tumors [70]. These barriers reduce the efficacy of anticancer therapies for GBM by acting as selectively permeable protective structures. Almost all low-molecular-weight drugs, as well as the majority of high-molecular-weight therapeutic agents—such as recombinant proteins, peptides, monoclonal antibodies, and viral vectors (including adenovirus-associated viruses)—are unable to cross the BBB [71,72]. The BBB is formed by the outermost layer of cerebral and spinal cord blood vessels and serves as a key immunological feature, acting as a structural and functional shield against bloodborne pathogens. A physiologically intact BBB consists of tight junctions supported by astrocytes and pericytes, enabling selective transport of oxygen and nutrients into the central nervous system while maintaining homeostasis [73,74,75,76]. Drug efflux proteins, such as P-glycoprotein (P-gp) and multidrug resistance proteins (MDRPs), further restrict drug penetration into the brain and reduce therapeutic accumulation, thereby contributing to treatment failure [77,78,79]. In GBM, the BBB becomes disrupted and exhibits extensive neoplastic infiltration. The development of GBM involves several steps, including tumor cell migration toward adjacent vasculature, detachment of astrocytic endfeet, and disruption of endothelial–basement membrane interactions via glioma-derived secretory factors [80]. The rapid formation of multilayered vasculature reflects BBB breakdown, as the formation of new vessels compromises tight junctions [81,82]. GBM patients often exhibit heterogeneous and regionally variable BBB dysfunction, with areas of intact barrier function remaining. This heterogeneity is sufficient to impair drug penetration into the tumor mass [83,84]. Vasogenic brain edema contributes to elevated intracranial pressure (ICP) and increased BBB permeability, representing a severe clinical complication of GBM [85]. Elevated ICP also reduces passive drug diffusion. Together, poor perfusion, high interstitial fluid pressure, heterogeneous BBB damage, and active drug resistance mechanisms result in reduced therapeutic efficacy in GBM [86]. The BBTB is a specialized interface separating the brain from systemic circulation, further preventing drug entry into the tumor site and complicating treatment [87]. The brain’s dense network of blood vessels, cellular components, and extracellular matrix impedes drug diffusion, making intratumoral distribution a major challenge in GBM treatment. Inadequate drug diffusion may prevent therapeutic agents from reaching all tumor regions. Moreover, the highly invasive nature of GBM makes it difficult for drugs to access the tumor’s full extent, as the malignancy tends to infiltrate distant brain regions [88]. Several strategies have been proposed to improve intratumoral drug diffusion, including the use of nanocarriers—such as liposomes or polymeric nanoparticles—to deliver drugs directly to the tumor site. These nanocarriers enhance drug diffusion through brain tissues and may increase treatment efficacy [89]. Despite progress, numerous challenges remain in optimizing drug delivery for effective GBM therapy. Further research is needed to elucidate the factors influencing drug diffusion in brain tissue and to develop more efficient delivery systems. Potential solutions include novel nanocarriers, combination therapies, and optimized delivery protocols. Additionally, the potential side effects and safety of these strategies must be carefully evaluated before clinical application. Cancer stem cells (CSCs) represent a small subpopulation within the tumor capable of self-renewal and differentiation into multiple cell types, contributing to tumor growth and recurrence. Their resistance to conventional therapies arises from various mechanisms, including drug efflux transporter expression, enhanced DNA repair capacity, and evasion of cell death pathways. One of the most significant challenges in targeting GBM CSCs is their anatomical location within the brain, which is protected by the BBB [90]. Several advanced delivery strategies have been developed to overcome this barrier, including convection-enhanced delivery, implantable pumps, and ultrasound-mediated delivery. Furthermore, the unique biological properties of CSCs—such as their resistance to apoptosis, capacity to form tumor spheres, and expression of drug-efflux transporters—render them highly refractory to standard chemotherapeutics [90]. To address this, nanotechnology-based approaches are being explored to selectively target CSCs and improve drug delivery efficacy. These strategies offer great promise for treating GBM and other malignancies characterized by resistance to chemotherapy and radiotherapy [91]. One of the most pressing challenges in GBM treatment is intratumoral heterogeneity, whereby distinct regions within the same tumor exhibit divergent molecular and genetic profiles. A major contributor to this phenomenon is the presence of cancer stem cells, which are thought to be resistant to many forms of therapy and are associated with tumor recurrence and progression [92]. Moreover, intratumoral heterogeneity may also arise from the brain’s unique microenvironment [93]. Recent advances in our understanding of GBM heterogeneity have led to the development of therapeutic strategies aimed at overcoming it. Personalized medicine, which tailors therapy to the patient’s molecular and genetic profile, has shown promise in improving outcomes. Additionally, combination therapies appear more effective than monotherapies in addressing tumor complexity. However, further research is required to fully characterize inter- and intratumoral heterogeneity and develop comprehensive treatments targeting all tumor subpopulations [94]. Beyond genetic and epigenetic alterations—including mutations, deletions, and gene amplifications—intratumoral heterogeneity is shaped by the brain microenvironment. Immune cells such as microglia and astrocytes contribute to tumor resistance and progression [95,96]. Despite these challenges, targeted therapies directed at specific genetic aberrations have demonstrated promising results in GBM treatment [97]. Immunotherapeutic approaches aimed at enhancing antitumor immune responses are also emerging as viable treatment options. Nonetheless, further investigation is necessary to develop therapies that comprehensively target all tumor cell populations. Recent research has highlighted the role of extracellular vesicles (EVs) in driving heterogeneity within GBM. EVs are small lipid bilayer-enclosed vesicles released into the extracellular space, capable of transferring biomolecules—including proteins, RNA, and DNA—between cells. Through the horizontal transfer of genetic and epigenetic material, EVs promote the emergence of genetically diverse tumor subclones [98]. Moreover, EVs can modulate gene expression in recipient cells by delivering RNA and DNA, thereby facilitating tumor progression and drug resistance [99,100]. EVs also mediate communication between glioma cells and the tumor microenvironment, interacting with immune cells such as microglia and astrocytes and modulating their behavior to alter the immune response [101]. Additionally, EVs interact with the extracellular matrix and influence the physical properties of the tumor microenvironment, further affecting tumor progression [102]. Therapeutic approaches targeting EV release or content have demonstrated preclinical efficacy in GBM models [101]. Furthermore, the use of EVs as delivery vehicles for therapeutic RNA or DNA offers a promising strategy for precise and efficient tumor targeting [103,104]. Both invasive and non-invasive strategies have been developed to overcome delivery barriers and improve CNS therapy. Invasive techniques include intrathecal administration via direct injection or catheterization [105], convection-enhanced delivery based on bulk flow [106], and the use of implants [107]. Non-invasive approaches involve chemical drug modification (e.g., lipidation) [108], carrier-mediated transcytosis [109], receptor-mediated transcytosis, and delivery using viral vectors or exosomes capable of traversing the BBB [110,111,112]. Additional methods include intranasal administration [111], BBB permeability modulation using hyperosmotic agents [113,114], and focused ultrasound techniques [115]. In the context of anticancer drug development for targeted delivery to glioma cells, modular design principles have been proposed [71,116]. The first module is a targeting moiety—small molecules or biological agents such as peptides, aptamers, proteins, or antibodies—which confers tumor specificity. The second component is an oncotoxic payload, such as a cytotoxic drug or radionuclide. The third module is a linker that connects the targeting moiety to the cytotoxic agent. Nevertheless, the long-term safety and efficacy of these approaches in GBM treatment require further evaluation [117].

### 2.3. Conventional Treatment Modalities

#### Description of Surgery, Radiotherapy, and Chemotherapy and Their Limitations

GBM management requires a multidisciplinary approach. The standard therapeutic regimen involves maximal safe surgical resection, followed by concomitant radiotherapy and temozolomide (TMZ)—an oral alkylating chemotherapeutic agent—and subsequent adjuvant chemotherapy with TMZ [118]. Because GBM tumors are often highly invasive and frequently located in eloquent brain areas responsible for speech, motor function, and sensory integration, achieving complete surgical resection is difficult. Infiltrating tumor cells inevitably remain in the surrounding brain tissue, leading to eventual disease progression or recurrence [119]. Advancements in surgical techniques and preoperative brain mapping have enabled more extensive resections while preserving function and quality of life [120]. The use of functional MRI, diffusion tensor imaging (DTI), and intraoperative tools such as ultrasound, CT, and direct cortical stimulation-guided MRI allows for multimodal neuronavigation that integrates patient-specific anatomical and functional data. Despite these technologies, differentiating between normal brain tissue and residual tumor remains a major challenge [121]. Limitations of these innovative technologies include high costs and the need for specialized equipment, trained operators, and operating facilities. Further studies are necessary to determine their clinical benefit before they can be adopted as the standard of care for all GBM patients. Despite surgical progress, the prognosis remains poor, with a median overall survival of approximately 15 months [122]. Beyond the extent of resection, other factors have been associated with improved overall survival. Patient age and Karnofsky Performance Status (KPS) are widely recognized prognostic indicators, with younger age and higher KPS correlating with longer survival. Tumors larger than 5–6 cm or those crossing the midline are linked to worse outcomes [123]. Supratentorial and cerebellar tumors, which are more amenable to surgical resection, are associated with better prognoses than tumors located in the brainstem or diencephalon [124]. Statistically significant improvements in overall survival have been observed following the initiation of aggressive multimodal therapy. Treatment of elderly patients is generally similar to that of younger cohorts, but performance status and comorbidities are crucial factors in individual decision-making [125]. With the exception of prolonged progression-free survival (but not overall survival) achieved with bevacizumab—a monoclonal antibody against vascular endothelial growth factor A (VEGF-A)—no pharmacological intervention has been definitively shown to alter the course of GBM [126]. Given the overexpression of VEGF-A, a key angiogenic driver, studies have evaluated the impact of bevacizumab in combination with standard radiochemotherapy and concurrent TMZ versus placebo. While bevacizumab did not improve overall survival, it significantly prolonged progression-free survival, preserved baseline quality of life, and maintained functional status [127]. However, bevacizumab is associated with an increased risk of severe hematologic and thromboembolic events. Current evidence suggests minimal benefit in elderly patients outside of clinical trial settings [128]. Radiotherapy combined with temozolomide chemotherapy remains the standard of care for elderly GBM patients with good Karnofsky Performance Status. Patients with tumors harboring an unmethylated MGMT promoter may still be considered for radiotherapy alone. TMZ monotherapy is an option for patients with MGMT promoter methylation when chemoradiation is not feasible [125]. MGMT promoter methylation silencing enhances the sensitivity of malignant cells to DNA-damaging alkylating agents. In contrast, the absence of MGMT silencing is associated with smaller and statistically insignificant differences in treatment outcomes between RT alone and RT plus TMZ [129]. The corticosteroid dexamethasone, at a dose of 2 to 4 mg, may alleviate treatment-associated symptoms, particularly by reducing radiation-induced edema. The dose should subsequently be tapered. The use of prophylactic antiepileptic drugs remains controversial; however, in patients who develop seizures, carbamazepine, phenobarbital, and phenytoin are generally avoided due to interactions with chemotherapeutic agents. Instead, levetiracetam, lacosamide, lamotrigine, and pregabalin are preferred owing to their more favorable interaction profiles [130].

## 3. Nanotechnology in the Treatment of GBM

### 3.1. Mechanisms of Nanoparticle Action in Cancer Therapy

Cancer is characterized by continuous evolution and unpredictable outcomes [131]. In recent years, significant advances have been made in clinical oncology, including patient-specific genetic/genomic profiling, immunotherapy, and more targeted therapeutic approaches [130]. The field of nanotechnology has introduced new hope into conventional methods for the treatment and diagnosis of gliomas, attributed to recent progress in bioengineering, improved drug availability, and the ability to specifically target cancer cells through their accumulation and entrapment [132]. Metal- and polymer-based nanomaterials are increasingly utilized in oncological therapy and diagnostics due to their small size, high surface area, distinctive structural properties, binding affinity, ability to penetrate cellular or tissue barriers, and prolonged half-life in circulation [133,134,135,136,137]. The high surface-to-volume ratio of nanoparticles enables the delivery of small biomolecules such as nucleic acids, proteins, and drugs to targeted sites, thereby enhancing the therapeutic efficacy [136]. One emerging strategy in cancer therapy is the use of drug carriers capable of circumventing cellular barriers. Extracellular vesicles (EVs), naturally occurring cell-derived particles, can serve as drug delivery vectors owing to their biocompatibility and inherent role in intercellular communication [131]. Due to their nanoscale dimensions, exosomes effectively traverse various tissue barriers while avoiding uptake by macrophages. This ability is attributed to their small size and limited expression of CD55 and CD59, which prevent opsonin activation and coagulation factors. Exosomes also exploit various surface proteins to facilitate intracellular uptake via endocytosis, rendering them highly efficient drug delivery vehicles. Encapsulation of drugs within exosomes protects therapeutic agents from circulating degradative enzymes, enhancing their delivery potential [138]. Historically, researchers have recognized the potential of organic nanomaterials such as liposomes in cancer treatment [139]. Using fusogenic liposomes (MFLs), it is possible to specifically deliver TPZ to distinct cellular compartments, such as the endoplasmic reticulum, followed by their incorporation into newly secreted exosomes released into the tumor microenvironment [138]. The utilization of glutathione, known for its antioxidant properties, as a targeting ligand involves its conjugation with PEGylated liposomes, improving brain tissue uptake via glutathione transporters [140]. A hallmark of cancer cells is metabolic reprogramming, which facilitates resistance to anticancer therapies. Glycogen metabolism plays a role in this metabolic shift under stress conditions such as hypoxia, glucose deprivation, or anticancer treatment. Consequently, targeting glycogen metabolic pathways represents a promising therapeutic strategy in oncology [141]. Nanoparticles possess immense potential in targeted cancer cell therapy and drug delivery, which is particularly significant in central nervous system oncology, where the blood–brain barrier (BBB) poses a substantial obstacle to drug delivery [133]. Nanoparticles can also be combined with chemotherapy (CDT), photodynamic therapy (PDT), and sonodynamic therapy (SDT) [134]. Nano-surgery in targeted therapy can be employed to remove residual microtumors or single cancer cells after macroscopically visible surgery in organs. These residual microtumors contribute to postoperative tumor recurrence. This approach uses various organic and inorganic nanoparticles for precise detection and removal of microtumors [133]. Light-responsive nanoparticles serve as powerful tools in nano-surgery and cancer treatment, demonstrating high efficacy as cytotoxic agents. These nanoparticles can be selectively targeted to specific cell types using appropriate recognition molecules [142]. Further advances in this strategy focus on tuning nanoparticle size relative to the proximity of the surgical field, overcoming BBB challenges, and optimizing nanoparticle conjugate functionalization to achieve maximal target site concentrations [143]. Nanovectors function as nanoparticles in the delivery and detection of anticancer drugs, thereby reducing toxic side effects. Nanovectors are classified into generations [144]. The first-generation targets cancer cell surface receptors nonspecifically [145]. The second generation focuses on active targeting, designed to identify and bind specific biomolecules expressed on cancer cells, incorporating high-affinity ligands and specific antigens on nanoparticle surfaces [146]. The third generation, currently under development, involves a multistage strategy [147]. Initially, biodegradable porous silicon microparticles are engineered to navigate the circulatory system and recognize disease-specific endothelium. Subsequent stages involve various nanoparticles loaded into the first-stage particles, released specifically at the tumor mass. These nanoparticles can traverse endothelial junctions and deliver diverse therapeutic and imaging payloads, representing a promising direction for future cancer therapies [148]. Nanomaterials constitute a continuously evolving family of materials with unique electrical, magnetic, and optical properties, which can be tailored to improve drug delivery and release within the tumor microenvironment [149]. Organic nanoparticles are established drug delivery systems with controlled release properties. Lipids, particularly phospholipid derivatives, can form physical micro- and nanostructures without chemical intervention [150]. Metal-organic frameworks (MOFs), comprising metal ions coordinated with organic linkers, represent a class of crystalline molecular materials used for the hierarchical integration of nanoparticles and/or biomolecules into a single structure for functionalization. These heterostructures, protected by MOFs, enhance the catalytic activity of nanoparticles without compromising the intracellular biological activity of biomolecules. Such structures can be combined with photothermal therapy, chemotherapy, radiotherapy, immunotherapy, and targeted therapy [151]. Among nanotechnological advances in cancer treatment is the development of nanomaterials that generate reactive oxygen species (ROS), thereby exacerbating tumor cell death via increased intratumoral oxidative stress. Various nanomaterials contribute to ROS production in cancer cells, disrupting redox homeostasis and inducing lipid peroxidation as well as oxidative damage to DNA and proteins [134]. Hyaluronic acid (HA) is a major component of the extracellular matrix (ECM), with elevated levels often observed in early tumorigenesis. HA is incorporated into various nanomaterials, including micelles, polymersomes, hydrogels, and inorganic nanoparticle formulations, and HA-based nanomaterials play a critical role in drug delivery systems [152]. HA is a common glycosaminoglycan (GAG) found in the brain, where it forms a hydrogel-like meshwork via interactions with other GAGs and proteoglycans [153]. HA is characterized by high water-binding capacity, non-toxicity, biodegradability, cellular compatibility, and lack of immunogenicity [154]. These exceptional properties have driven the development of HA-based nanomaterials for diverse biomedical applications, such as drug delivery systems (DDS) and molecular imaging [152]. Numerous cancer cells, including those in GBM tumors, exhibit overexpression of HA-binding receptors such as CD44, LYVE-1, and RHAMM [155]. Multiple studies highlight HA overexpression in GBM and its influence on tumor progression [153]. The functionalization of nanoparticles (NPs) with active groups like HA facilitates active targeting, enhancing selectivity toward cancer cells [156]. Metal sulfide nanomaterials (MeSNs) are known for their enhanced biocompatibility and unique features in cancer therapy, including Fenton catalysis, light conversion, radiation enhancement, and immune system activation. Intact MeSNs effectively convert energy for phototherapy and radiotherapy, conferring synergistic antitumor properties that represent a significant advantage over other nanotherapeutics. The therapeutic efficacy of MeSNs depends on intrinsic factors such as tumor site accumulation. Despite these promising anticancer effects, MeSN applications remain in early developmental stages [157]. Polymers such as polylactic acid (PLA), polylactic-co-glycolic acid (PLGA), and poly(ε-caprolactone) (PCL) have the capacity to encapsulate or adsorb drug compounds. With proper functionalization, they can enhance the delivery of both hydrophobic and hydrophilic small-molecule drugs to designated target sites [158]. The blood–brain barrier (BBB) poses a challenge due to its selective permeability. The use of nanotransporters equipped with targeting moieties offers a potential strategy to penetrate glioma cores. These moieties can bind membrane receptors present both in tumor-infiltrated and healthy BBB, facilitating nanodrug transport. Malignant glioma, with its heterogeneous cellular populations, contains cancer stem cells responsible for treatment resistance [159]. Poor penetration often compromises therapeutic efficacy [133]. Conversely, in targeted drug delivery, therapeutic agents accumulate at the target site via circulation. Based on delivery mechanisms, targeted therapy is classified into two main categories: (1) Passive targeting, where therapeutic particles are sequestered by physiological phenomena such as the enhanced permeability and retention (EPR) effect in tumor tissue; and (2) Active targeting, where the therapeutic agent is modified by a specific ligand whose receptor is highly expressed at the target site. Combining both approaches, such as particle modification with certain morphological features, results in superior delivery compared to either approach alone. Thus, employing passive targeting strategies in GBM, inherently limited by BBB inaccessibility, would render treatment ineffective [160]. Through a function termed ‘controlled release reservoir,’ nanoparticles have demonstrated considerable efficacy in releasing therapeutics in proximity to target sites. However, prior to clinical application, certain criteria such as biocompatibility must be met, since the primary goal of targeted therapy is to avoid adverse events induced by conventional treatment [161]. Surface charge can influence binding to endothelial cells and transcytosis, making both cationic and neutral porous nanoparticles potential candidates for brain drug delivery. A common method for nanoparticle surface modification is PEGylation, involving conjugation with polyethylene glycol (PEG). PEGylation has been shown to reduce opsonization, resulting in decreased uptake by the reticuloendothelial system (RES) and prolonged circulation time of PEGylated nanoparticles [162]. PEGylation significantly reduces plasma protein adsorption compared to uncoated nanoparticles, with variations in protein amounts over time. Nanospheres exhibited extended blood circulation and reduced hepatic accumulation, dependent on the molecular weight and surface density of PEG coatings. They can also be lyophilized and reconstituted in aqueous solutions, demonstrating good storage stability. This enables the customization of ‘optimal’ polymers for specific therapeutic applications [163]. The application of nanotechnology in GBM treatment aims to enhance targeting precision, increase bioavailability, and minimize adverse effects by improving drug internalization into cells while reducing off-target accumulation in organs [164].

### 3.2. Types of Nanomaterials in GBM Therapy

**Figure 3:**
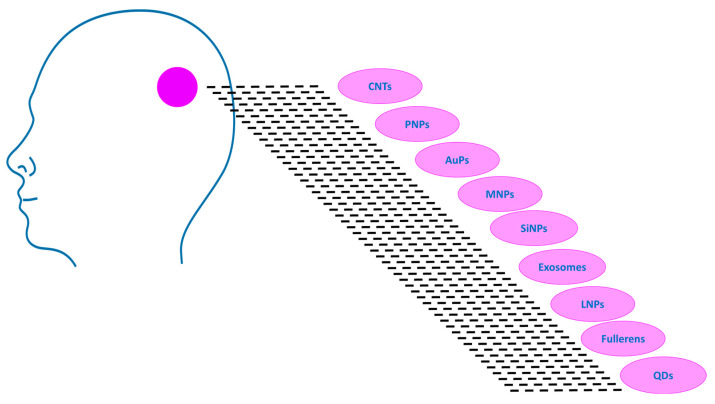
The perspective of various NPs in GBM research. Abbreviations are: CNTs (carbon nanotubes made of carbon with a diameter in the nanometre range -nanoscale); PNPs (polymeric nanoparticles); AuPs (gold particles); MNPs (metal nanoparticles); SiPNs (silica nanoparticles); exosomes (mediators of intercellular communication between GBM cells and surrounding cells, such as immune cells); LNPs (lipid nanoparticles); QDs (quantum dots).

We describe the major types of nanoparticles being used in research as potential candidates for the GBM treatment (Figure 3).

#### 3.2.1. Lipid Nanoparticles: Liposomes, Micelles, and Their Application in Drug Delivery to Brain Tumors

Lipid nanoparticles (LNPs) were developed to overcome all the limitations associated with polymeric nanoparticles, such as high production costs, high toxicity related to the use of solvents during their synthesis, and polymer-related allergies [165]. The first model of lipid-based nanoparticles was liposomes, introduced in 1965. The first nanoparticle used in medicine was the liposomal nanoformulation of amphotericin B, approved in Europe in 1990. A few years later, the FDA approved a PEGylated liposomal formulation of doxorubicin for cancer treatment. Despite the unique advantages of liposomes, such as high biocompatibility, low toxicity, lack of immunogenicity, and biodegradability, their applications were limited due to certain drawbacks, including the susceptibility of phospholipids in liposomes to oxidation and hydrolysis, poor stability, short shelf life, low encapsulation efficiency, and high production costs [166]. In 1990, solid lipid nanoparticles (SLNs) and nanostructured lipid carriers (NLCs) were identified as alternative drug carriers to classical nanocarriers like polymeric nanoparticles, liposomes, and emulsions. SLNs and NLCs demonstrated higher stability and better release profiles compared to liposomes, as well as a better safety profile compared to polymeric nanoparticles that do not use organic solvents [167,168,169]. SLNs and NLCs promote drug delivery to target cells through various mechanisms, including active and passive targeting. In passive mechanisms, SLNs and NLCs exploit specific properties of the tumor microenvironment to enhance drug delivery, relying on the enhanced permeability and retention (EPR) effect. However, in active mechanisms, the surface of SLNs and NLCs is modified to recognize transporters or receptors that are overexpressed on target cells, which sometimes leads to selective targeting and minimization of side effects. Additionally, SLNs and NLCs possess an inherent ability to cross the blood–brain barrier (BBB) and serve as suitable carriers for a broad spectrum of GBM therapies, including large molecules, genes, oligonucleotides, siRNA, and enzymes. These features make SLNs and NLCs among the best candidates for drug delivery in the treatment of GBM and other brain diseases [170]. SLNs also have some drawbacks, such as unexpected gelation tendency, low encapsulation efficiency (EE), and unpredictable release of the embedded therapy due to recrystallization of the solid lipid, which hinders sustained drug retention. These limitations were the main reason behind the idea of introducing liquid lipid into the SLN formula, creating so-called NLCs [168]. Various nanoparticle design strategies have been tested to transform simple nanoparticles for drug delivery, without considering many aspects related to smart nanocarriers with optimized properties. A smart drug delivery system is a carrier that can deliver drugs or therapies to target cells without affecting healthy tissues, has a specific desired release profile, can avoid clearance by the immune system, and can ultimately be used for co-delivery of drugs with another substance such as genetic material, diagnostic agents, or, in some cases, combined chemotherapy [171]. Different modifications of SLNs and NLCs have been applied to transform them from conventional drug carriers into smart drug delivery systems capable of overcoming all barriers and challenges associated with GBM treatment. SLNs and NLCs possess some ability to cross the BBB due to their lipid nature. This type of strategy often exploits two features of the BBB, namely: the receptor-mediated transcytosis (RMT) pathway using Trojan-Horse molecules attached to the nanocarriers or the adsorptive-mediated transcytosis (AMT) pathway using cationic nanoparticles [81,172]. Studies have shown that conjugation of angiopep-2 on the surface of nanoparticles enhances drug delivery to GBM cells because it can bind to lipoprotein receptor-related protein 1 (LRP1) on the BBB. Regarding the use of the AMT system to increase BBB permeability, various approaches to develop positively charged LNPs can be employed. These include the use of cationized proteins such as albumin, cationic lipids such as stearylamine, and cell-penetrating peptides (CPPs) such as protamine [81,173]. Multidrug resistance (MDR) is a major obstacle to effective chemotherapy in GBM treatment. MDR is typically mediated by three proteins: P-glycoprotein (P-gp), breast cancer resistance protein (BCRP), and multidrug resistance-associated protein 1 (MRP-1). Various nonionic surfactants have demonstrated the ability to reverse MDR mechanisms. For example, Pluronic P85 (a block copolymer surfactant) can sensitize MDR cancer cells to various chemotherapeutic drugs by depleting ATP [174]. Additionally, nonionic surfactants such as Brij exhibit inhibitory effects against P-gp via similar mechanisms. Another surfactant, TPGS 1000 (D-alpha-tocopheryl polyethylene glycol 1000 succinate), bypasses P-gp efflux by inhibiting ATP activity without acting as a P-gp substrate or competitive inhibitor [175]. Polyethylene glycol (PEG) derivatives, such as PEG stearate and PEGylated glyceride fatty acid esters, have also been proposed as P-gp efflux inhibitors [176]. Surface modification with PEG has been shown to improve pharmacokinetics and brain delivery by promoting ionic interactions between core and shell, resulting in sustained drug release [177]. One successful approach to targeting efflux pumps combines multiple strategies in a single nanoparticle carrier. Other efflux inhibition strategies employed SLNs and NLCs for co-delivery of two drugs: an efflux pump inhibitor and a chemotherapeutic or gene therapy agent to silence efflux genes. Since P-gp efflux limits doxorubicin penetration into the brain for cancer treatment, folic acid-modified SLNs (SLA) were developed to co-deliver doxorubicin and ketoconazole (a P-gp inhibitor) to the brain [178]. Studies assessing SLA in brain endothelial cells revealed that folic acid-modified SLNs enhanced brain penetration of both drugs. Gene silencing of P-gp represents another strategy to improve drug delivery to tumor cells. Currently, limited studies explore SLN and NLC use in gene therapy for multidrug-resistant cancers, with recent research focusing more on inorganic or polymeric nanoparticles as carriers [179]. Recent efforts have concentrated on surface engineering of lipid carriers by conjugating various ligands such as peptides, proteins, carbohydrates, monoclonal antibodies, and small molecules. These ligands recognize overexpressed targets on tumor cell surfaces, promoting accumulation in the tumor microenvironment and minimizing nonspecific distribution. Other modification strategies exploit tumor microenvironment features such as acidic pH, hypoxia, and enzymatic hydrolysis by developing stimuli-responsive lipid-based nanoparticles [180]. Folic acid is a widely used small molecule ligand for targeted therapy, as the folate receptor is highly expressed in various cancers, including lung, colorectal, and GBM. Cetuximab, a monoclonal antibody, binds with high affinity to the epidermal growth factor receptor (EGFR), which is often dysregulated in GBM cells. To achieve more targeted brain tumor therapy, researchers [181,182] conjugated cationic SLNs with anti-EGFR monoclonal antibody (cetuximab) to deliver carmustine to malignant GBM cells. EGFR-targeted SLNs specifically bound to EGFR on U87MG cells, enhancing carmustine transport and reducing the required dose. Peptides have also become effective targeting ligands due to advantages like small size, high stability, low immunogenicity, and high selectivity [183]. The RGD peptide (arginine–glycine–aspartic acid) is widely used to target neoangiogenesis by binding integrin receptors overexpressed on GBM cells and tumor-associated endothelial cells. Endogenous proteins such as transferrin, lactoferrin (Lf), and interleukin-13 (IL-13) can be exploited as targeting ligands due to their selective receptor binding and endocytosis mediation [184]. Lactoferrin, a cationic iron-binding glycoprotein from the transferrin family, has receptors expressed on BBB endothelial cells and GBM cells. To enhance SLN targeting in brain tumor therapy, researchers [185] prepared Lf-conjugated SLNs for efficient docetaxel delivery to the brain. Receptor saturation assays and brain distribution studies confirmed improved targeting efficacy and brain uptake with Lf-docetaxel SLNs. Among carbohydrate ligands, hyaluronic acid is a natural polysaccharide with high affinity for CD44, overexpressed in various tumors, including melanoma, breast, colorectal, and brain cancers. Researchers [186] employed hyaluronic acid as an active targeting ligand to develop liposomal drug delivery systems capable of distinguishing malignant GBM cells from healthy brain cells. Cellular uptake assays in primary astrocytes, microglia, and GBM cells demonstrated selective targeting of tumor cells due to higher CD44 expression. Hyaluronic acid has also been used for targeted SLN and NLC delivery against various cancers [187,188]. Matrix metalloproteinases (MMPs) are a group of proteolytic enzymes elevated in GBM and other tumors. Present as catalytic markers in the tumor microenvironment, MMPs have long been associated with cancer cell behaviors such as migration, invasion, apoptosis, and differentiation. Thus, MMPs can serve as enzymatic triggers for nanoparticle activation [189]. Furthermore, PEGylated pH-sensitive nanoparticles have been developed for targeted therapies [190]. Hypoxia is a prominent feature of tumor tissue, with redox potential differing markedly from that of normal tissue. This difference enables the design of hypoxia-sensitive drug delivery systems. Studies have shown glutathione levels to be 100–1000 times higher in tumors than in blood and 100 times higher than in normal tissues. Hence, nanoparticles incorporating disulfide bonds can maintain structural integrity under normal conditions but undergo bond reduction to thiols in glutathione-rich tumor cells, resulting in nanoparticle destabilization and payload release [191]. The direct anatomical connection between the nasal cavity and the central nervous system (CNS) renders nasal-to-brain drug delivery a highly promising route, offering several advantages that overcome challenges related to conventional administration routes. These include non-invasiveness, ease of administration, rapid onset of action, large absorption area, reduced enzymatic activity, and avoidance of first-pass hepatic metabolism. These benefits have driven an increasing number of products utilizing the nasal route for CNS drug delivery [192]. Among various nanoparticle carriers, lipid nanoparticles (LNPs), including solid lipid nanoparticles (SLN) and nanostructured lipid carriers (NLC), administered via the nasal route, have demonstrated efficacy as drug delivery systems for central nervous system (CNS) disorders such as neurodegenerative diseases and brain tumors. The mechanism by which nanoparticles enhance nose-to-brain drug delivery involves interaction with the mucosal layer, followed by release of the encapsulated drug into mucosal cells or penetration through the mucus to be internalized by neurons, which subsequently transport the drug via axonal pathways to the brain for release. Effective interaction of nanoparticles with this biological environment is dictated by physicochemical characteristics of the carrier, including composition, size, and surface charge. For instance, mucociliary clearance represents a significant barrier that limits the residence time of substances administered intranasally [193]. Thus, surface coating of LNPs with agents such as chitosan, hyaluronic acid, or low molecular weight pectin can prolong retention time in the olfactory region [194]. Moreover, mucins within the mucus layer contain elevated levels of sialic acid and sulfate residues, imparting a negative charge and contributing to the layer’s rigidity. Consequently, utilizing nanoparticles modified to possess a cationic surface enhances electrostatic interactions with the mucosal membrane, extending contact time. However, prolonged interaction of formulations with the nasal mucosa may induce undesirable side effects such as epithelial irritation and cytotoxicity. Protection of the primary olfactory nerves and the sense of smell from damage by cytotoxic drugs must be carefully considered [195]. SLN and NLC are described as superior candidates for targeting GBM multiforme (GBM) via the nasal route owing to their high biocompatibility, low toxicity, and facile surface functionalization. Intranasal nanoparticles can also be functionalized with lectins—glycoproteins derived from plants like tomato and wheat germ—with selective affinity for glycan residues on biological surfaces, potentially enhancing adhesion and targeted delivery in the nasal epithelium [196]. However, lectin-functionalized nanoparticles targeting the nasal epithelium may pose challenges due to their toxicity to mammalian cells [197]. Despite the promising literature reports on the use of SLNs and NLCs for GBM treatment—with diverse drugs, materials, and functionalization strategies—no such carriers have yet been successfully developed by pharmaceutical companies or brought to market. Therefore, it is crucial to critically reassess all current approaches in SLN and NLC design. Greater efforts are needed to optimize scalable manufacturing techniques and ensure reproducible production of nanocarriers. Additionally, there is a pressing demand for further investigations elucidating the safety concerns related to nanoparticle size and their potential to penetrate cellular membranes and interact with various biological systems. These issues can be addressed through in vivo studies aimed at predicting nanoparticle toxicity across organs. For nasal-to-brain delivery nanoformulations, long-term pulmonary toxicity studies are essential to confirm safety. Considering these challenges, significant advancements in SLN and NLC technology hold promise as intelligent drug delivery systems for the treatment of malignant GBM [198].

#### 3.2.2. Metallic and Carbon Nanoparticles: Gold, Iron, Carbon Nanotubes–Their Properties and Therapeutic Potential

Inorganic nanoparticles possess several critical features, including the ability to tailor morphology and nanostructure, facile functionalization, notable physiological stability, and distinctive physicochemical properties—optical, electrical, acoustic, and magnetic attributes—that distinguish them from conventional organic or polymer-based counterparts [199]. The latest magnetic nanoparticles (MNPs) exhibit many advantageous physical and chemical properties, such as wider operational temperature ranges, reduced sizes, lower toxicity, simpler preparation methods, and decreased production costs. These characteristics endow them with promising prospects across various medical applications, including use as probes in medical imaging and as carriers in targeted drug delivery systems [200]. Magnetic nanoparticle-based systems generally occur in two primary forms: inorganic cores coated with polymer surfaces and nanoparticles embedded with inorganic crystals [201]. Typically composed of pure metals (Fe, Co, Ni, and some rare earth metals) or metal–polymer composites, these nanoparticles provide enhanced magnetic moments and high surface-to-volume ratios, making them attractive for hyperthermia therapy in cancer treatment as well as targeted drug delivery. They also serve as contrast agents in magnetic resonance imaging (MRI) and enhance the sensitivity of biosensors and diagnostic tools [202]. Nanomagnetic materials are characterized by a large specific surface area and the capability to transport various small molecules, proteins, RNA, and others. The magnetic properties of metal-based nanoparticles facilitate their enrichment, separation, movement, and precise positioning [94,203,204]. MNPs also exhibit a magnetocaloric effect under high-frequency magnetic fields, which can induce indirect elimination of cancer cells [203]. Currently, magnetic nanopowders (MNPs) are widely applied in medicine, including drug delivery [200]. MNPs are extensively used in magnetic hyperthermia, MRI imaging, photodynamic therapy (PDT), and photothermal therapy (PTT). Surface coatings on MNPs aim to enhance colloidal stability, allow therapeutic payload attachment, and regulate the pharmacokinetics and pharmacodynamics of MNPs [205]. As drug delivery systems, MNPs can be loaded with anticancer agents such as curcumin, temozolomide (TMZ), and paclitaxel (PTX), leading to suppression of GBM cell proliferation [206]. The most commonly synthesized iron oxide nanoparticles (γ-Fe_2_O_3_ or Fe_3_O_4_) are prevalent in cancer therapeutics due to their magnetic responsiveness and good patient tolerability [205]. Their therapeutic efficacy depends on temperature [207,208]. The size and surface functionality of these nanoparticles play critical roles in pharmaceutical applications [209]. Particles larger than 200 nm are readily filtered by the reticuloendothelial system, while particles smaller than 8 nm are rapidly cleared via renal excretion, shortening their circulation time [210]. Particles sized between 10 and 40 nm exhibit the longest blood retention and can be stabilized to a target size through application of an external magnetic field, reducing required dosage and potential side effects. Therapeutic success largely depends on the composition of the external coating layer; therefore, polymer layers, capsules, particles, or vesicles are proposed as outer layers. Surface modifications are achieved using organic polymers and inorganic metals or oxides [208,211]. Given their reactive surfaces and ability to cross biological barriers, they are among the nanoparticles selected for clinical applications [210]. In this context, anticancer agents such as doxorubicin, docetaxel, 5-fluorouracil, gemcitabine, and methotrexate can be encapsulated within inorganic magnetic nanoparticles [211,212]. Studies show that inorganic nanoparticles can stimulate T-lymphocyte-mediated immune responses against tumors. Upon accumulation at tumor sites, they can generate heat under external alternating magnetic fields, killing cancer cells and enhancing immune function within the tumor microenvironment by releasing pro-inflammatory cytokines [213]. They may activate NADPH oxidases, induce reactive oxygen species (ROS) production, and promote redox homeostasis imbalance, rendering them effective tools for malignant cell eradication [214]. Gold nanoparticles (GNPs) have been utilized as specific drug carriers for tumors, imaging agents, radiosensitizers, and antiangiogenic agents due to their easily controlled and modifiable shape, size, and surface chemistry, along with biocompatibility and lower cytotoxicity [215]. In vitro studies demonstrate GNP cytotoxicity in cells via oxidative stress induction. Apoptosis induced by oxidative stress is a key mechanism of GNP toxicity. ROS can disrupt the balance between oxidizing and antioxidative processes within cells [216,217]. Recent studies show that the size-dependent cytotoxicity of GNPs increases with deeper penetration into the target tissue [216]. U2-AuNP, a gold-based nanopowder, was investigated for its effect on cell lines and antitumor activity in mice bearing GBM. Results indicated U2-AuNP inhibited proliferation and invasion of U87-EGFRvIII cell lines and blocked EGFR-related signaling pathways, preventing DNA damage repair in GBM cells [218]. Carbon nanomaterials (CNMs), such as graphene, carbon nanotubes (CNTs), and quantum dots, represent a class of nanomaterials with high capacity for targeting cancer cells [219]. CNTs, formed from cylindrical graphite sheets, are highly stable, biocompatible, and non-immunogenic, offering significant value in targeted drug delivery [220,221]. ROS generation is a major mechanism underlying the anticancer activity of carbon nanoparticles. Additionally, their large surface area adsorbs other chemicals, which, upon biotransformation, can be oxidized to active redox quinones. Their anticancer effects are attributed to matrix metalloproteinase (MMP) regulation, inhibiting tumor metastasis, and enhancing antitumor immunity via ROS generation and activation of Toll-like receptors (TLRs) in phagocytes [214]. Use of CNTs for targeted tumor hyperthermia is under investigation in glioma treatment. These nanoparticles convert near-infrared light into heat, selectively heating and destroying cancer cells [222]. Quantum dots (QDs), distinguished by their size and unique optical and electronic properties, may revolutionize glioma treatment. Graphene quantum dots (GQDs) have shown promise due to their biocompatibility and distinctive photophysical attributes [223]. Studies demonstrated their ability to cross the blood–brain barrier, a key challenge in brain cancer therapy. In 3D glioma spheroid models, surface-functionalized GQDs not only increased membrane fluidity and intracellular uptake but also synergized with chemotherapeutics such as doxorubicin and temozolomide at subtherapeutic doses. A novel therapeutic strategy, photothermal therapy (PTT), where GQDs absorb near-infrared light and convert it to heat, enhances membrane permeability and potentiates chemotherapy effects. Combined PTT and chemotherapy significantly reduced tumor growth and viability, highlighting GQDs’ potential to mitigate side effects and modulate immune responses, improving patient quality of life [224]. These findings were complemented by the implementation of INSIDIA 2.0 software for image analysis, enabling high-throughput and high-content quantitative in vitro assessment of 3D tumor spheroids. This tool provided key insights, allowing researchers to non-invasively and rapidly quantify cell death, with results showing decreased spheroid surface area and formation of dense, uniform spheroid cores upon GQD photothermal therapy in U87 glioma spheroids [225]. Expanding quantum dot applications, researchers explored neodymium ion-coordinated black phosphorus quantum dots (BPNd) for targeted glioma therapy. BPNd demonstrated excellent performance in the second near-infrared window (NIR-II) fluorescence imaging and X-ray-induced photodynamic therapy. The study highlighted optoelectronic switching between BPNd and Nd3+ ions, enabling precise intracranial glioma growth monitoring via NIR-II fluorescence imaging and effective inhibition through targeted X-ray-triggered photodynamic therapy. Ultraminiature BPNd, combined with high loading capacity, facilitated blood–brain barrier penetration, representing a promising path for precise and effective glioma therapeutic strategies [226]. Collectively, these studies underscore the transformative potential of quantum dots in advancing targeted and effective GBM therapies. Whether through multifaceted GQD applications in photothermal therapy and chemotherapy or innovative BPNd use in NIR-II fluorescence imaging and photodynamic therapy, quantum dots hold promising prospects for shaping GBM treatment. These advances not only enhance therapeutic outcomes but also contribute to side effect reduction and patient well-being improvement [227].

In conclusion, nano-based technology holds immense promise for revolutionizing GBM treatment by offering innovative solutions for drug delivery, imaging, and targeting, ultimately leading to improved patient outcomes.

Radiation therapy remains a critical component of many cancer treatment plans, and when combined with other therapies such as surgery, chemotherapy, targeted therapies, and immunotherapy, it can significantly improve treatment outcomes. Combining radiotherapy and chemotherapy is a standard procedure in the radical treatment of many cancers. The objective of chemoradiotherapy is to increase loco-regional control, to reduce the risk of distant metastases, and to prolong survival, and thus to improve treatment efficiency with less mutilating therapies.

#### 3.2.3. Polymeric Nanoparticles: Applications in Controlled Drug Release and Precision Therapy

Polymeric nanoparticles (NPs) represent a promising drug delivery platform in the treatment of GBM multiforme (GBM). These nanocarriers are fabricated from biodegradable polymers that enable controlled and sustained release of therapeutic agents, which is crucial for maintaining effective drug concentrations within the tumor over extended periods [228]. Polymeric NPs significantly enhance drug bioavailability by increasing stability and reducing degradation [229,230]. Moreover, surface modifications with targeting ligands—such as antibodies, peptides, or small molecules—allow for specific binding to tumor cells, thereby increasing drug accumulation in tumors while minimizing toxicity to healthy tissues [231]. Physical and chemical modifications further improve their ability to cross the blood–brain barrier (BBB), enhancing drug distribution and concentration in brain tissues [232]. Due to their excellent biocompatibility and tunable properties, polymeric nanoparticles have been extensively studied in GBM therapy. Various classes of polymeric nanoparticles—including micelles, dendritic polymers, polymeric vesicles, hydrogels, and metal-organic frameworks (MOFs)—differ in structure, composition, and functional capabilities, offering distinct advantages for targeted therapeutic applications. Micelles are characterized by a hydrophobic core and hydrophilic shell, conferring amphiphilic properties [233]. This allows micelles to encapsulate hydrophobic drugs and release them under specific conditions, such as pH, temperature, or enzymatic activity [234]. Such controlled release enhances drug efficacy at the target site. Additionally, micelles improve the solubility and targeted delivery of hydrophobic drugs [235]. Studies indicate that polymeric micelles facilitate effective drug release and cellular uptake, improving therapeutic outcomes [236]. Micelle properties—such as size, shape, and surface characteristics—can be tailored via polymer selection and synthesis conditions to suit various applications [237]. Beyond drug delivery, micelles are also employed to transport imaging agents or diagnostic reagents for biomedical imaging and targeted drug delivery [238]. For instance, pH-sensitive micelles are amphiphilic polymers undergoing structural changes in response to pH variations [239]. Ionizable groups on polymer chains protonate or deprotonate under different pH, causing micelle disassembly or reassembly, thereby controlling drug release [240]. This mechanism enables drug release in pathological environments, such as tumor microenvironments (TME), enhancing therapeutic effects while minimizing damage to healthy cells. Reduction-sensitive micelles comprise polymeric surfactants designed to release drugs through reduction reactions under specific conditions [241]. In the presence of reductants, these microspheres undergo structural changes, facilitating controlled release [242]. By concentrating drugs at target sites, they enhance therapeutic efficacy and reduce side effects [243]. Owing to high biocompatibility and regulated release properties, reduction-sensitive microspheres offer an effective targeted drug delivery system. Photosensitive micelles form via spontaneous self-assembly of zwitterionic polymers responsive to specific light wavelengths [244]. Containing both hydrophilic and hydrophobic segments, these micelles encapsulate drugs and release them upon light exposure. Photosensitizers within micelles initiate photochemical reactions, resulting in drug release or micelle depolymerization [245]. Light intensity and wavelength can be adjusted to precisely control release rates, minimizing potential harm to healthy tissues [246]. Coupled with imaging techniques, photosensitive micelles facilitate real-time monitoring of drug release and therapeutic efficacy. Dendritic polymers are highly branched synthetic structures produced via polymerization techniques, including dendrimers and dendritic polymer networks [247]. Characterized by repetitive branching units, these polymers form highly symmetrical and versatile platforms with broad applications in drug delivery, gene transfer, imaging, and detection [234]. As drug carriers, dendritic polymers deliver therapeutics directly to target cells or tissues, increasing efficacy while minimizing adverse effects. Their surfaces can be functionalized with targeting groups, and their small size and surface properties promote efficient cellular uptake via endocytosis [248]. Additionally, their porous structures enable controlled drug release in response to environmental stimuli such as pH fluctuations or enzymatic activity, contributing to therapeutic success [249]. Their highly branched architecture provides a large surface area for drug or gene binding, forming stable carriers that improve solubility and bioavailability while preserving therapeutic activity in vivo [250]. These polymers can also combine therapeutic and imaging functions in a single carrier, enhancing targeting capabilities. Convergent dendritic polymers, synthesized via convergent methods, exhibit similarly highly branched structures [251]. Compared to linear or crosslinked polymers, these dendritic polymers possess unique spatial configurations and functional versatility [252]. Their surfaces can be modified with targeting groups to facilitate binding to specific cells or tissues, enabling controlled release triggered by environmental factors (e.g., pH or temperature changes). The dendritic structure enhances membrane penetration and drug endocytosis [253]. Surface modifications further improve drug delivery efficiency and bioavailability compared to traditional carriers [254]. Polymeric vesicles are amphiphilic polymer-based structures resembling liposomes [255]. Their formation involves selecting appropriate polymers and employing methods like solvent evaporation or spontaneous assembly to generate stable vesicles [234]. This structural stability maintains drug efficacy in vivo by protecting against degradation [256]. Polymeric vesicles enter cells via endocytosis to release their contents intracellularly. Surface modifications enable targeted delivery to specific cells or tissues, enhancing therapeutic efficacy [257]. Besides drug delivery, polymeric vesicles serve as gene carriers for DNA or RNA and vaccine delivery vehicles to boost immune responses. Temperature-sensitive polymeric vesicles respond to temperature changes by altering physical or chemical properties [258]. Typically, conformational changes in polymer chains at a critical temperature affect solubility and biomolecular interactions [259]. For example, poly(N-isopropylacrylamide) (PNIPAM) is a widely used thermosensitive polymer that transitions from hydrophilic to hydrophobic above its lower critical solution temperature, enabling controlled drug release [260]. Drugs can be loaded below this temperature and released upon heating when the polymer collapses [261]. This rapid thermal response allows precise control over drug release rates, enhancing therapeutic effects and reducing side effects in non-target tissues [262]. Thermosensitive polymers are also applied in bioimaging and sensor development. pH-sensitive polymeric vesicles alter their physical or chemical properties in response to environmental pH changes [263]. Composed of polymers bearing acidic or basic groups, these vesicles exhibit reversible solubility or aggregation under defined pH conditions, facilitating controlled drug release [263]. At specific pH levels, solubility changes trigger release in target environments such as acidic tumor microenvironments (TMEs) [264]. Hydrogels consist of hydrophilic polymers forming three-dimensional networks in water, imparting biocompatibility, flexibility, and moisture retention [265]. Their structural stability arises from mechanisms including physical adsorption, chemical bonding, and reversible swelling or contraction [266]. These properties make hydrogels highly suitable for various biomedical applications, including drug delivery and tissue engineering [267]. In drug delivery, hydrogels effectively control release rates, enhancing bioavailability [268]. Acting as scaffolds, they support cell growth and tissue regeneration, while their high hydration and conductivity make them ideal for biosensor development [268]. Thermosensitive hydrogels undergo changes in hydration state and structure near critical solution temperatures, allowing water uptake or release as needed [269]. As temperature rises, phase transitions or chemical interactions condense polymer chains, expelling water and reducing volume [270]. Polymer composition and crosslink density can be tuned to regulate these properties. Biocompatible and non-toxic, thermosensitive hydrogels are widely used to modulate drug release rates and facilitate temperature-triggered delivery [271]. pH-sensitive hydrogels respond to environmental pH changes by altering physical and chemical properties [261]. Functional groups (e.g., acidic or basic moieties) on polymer chains ionize or deionize at different pH, affecting hydrophilicity and swelling, which in turn modulates drug release kinetics [267]. Sensitivity can be precisely adjusted by modifying the polymer chemical structure and composition. These hydrogels enable controlled drug release in specific pH environments (e.g., TMEs or gastrointestinal tract) and find broad applications in tissue engineering and biosensors [272]. Metal-organic frameworks (MOFs) are porous materials constructed by the coordination of metal ions or clusters with organic ligands, resulting in large surface areas and tunable pore sizes suitable for diverse applications [273]. Their extensive surface area provides numerous active sites, enhancing adsorption capacity and improving drug loading and release efficiency [274]. The porous structure and surface functional groups facilitate interactions with drug molecules, enabling efficient adsorption, release, and targeted delivery—key attributes for drug delivery systems [275]. Their biocompatibility and low toxicity further increase potential medical applications [276]. MOFs are highly ordered and customizable, with pore sizes and chemical environments optimized by selecting appropriate metals and ligands for specific uses [277]. Beyond drug delivery, MOFs are employed for gas capture (e.g., CO_2_ and H_2_ storage) and as catalysts or catalyst carriers, improving reaction selectivity and activity [278]. Most MOFs exhibit stable physicochemical properties under humid or high-temperature conditions and can be synthesized from renewable materials, promoting sustainability [279]. Stimuli-responsive MOFs, composed of metal ions and organic ligands, offer tunable porosity and adapt their properties in response to external stimuli [280]. For example, pH changes can protonate organic ligands, modifying pore size and affecting drug release rates. In biomedical applications, these MOFs enable targeted, controlled drug delivery, reducing side effects [281].

## 4. Examples of Studies and Clinical Trials

### 4.1. Preclinical Studies

#### 4.1.1. In Vitro Studies

##### PLGA-PEG Nanoparticles with antagomiR-21 and antagomiR-10b

cRGD-functionalized green PLGA-PEG nanoparticles demonstrate enhanced uptake by U87MG and Ln229 glioma cell lines. When combined with a low dose of temozolomide (TMZ), they effectively induce apoptosis and cause G_2_/M cell cycle arrest through the upregulation of PTEN, PDCD4, and CASP proteins [282].

##### PTX–Oligo(p-phenylenevinylene) Nanoformulation

This formulation crosses the blood–brain barrier (BBB) at approximately 10% efficiency and accumulates in tumor tissue. It induces a higher apoptosis rate in U87MG and U343 cell lines and inhibits tumor growth in vivo by more than 50%, compared to a 26% reduction observed with free paclitaxel (PTX) [283].

##### Chitosan-PLGA for Intranasal Application

Mucoadhesive nanoformulations containing chitosan (CHC) and cetuximab reduce viability to approximately 5% in SW1088 and U251 models [283].

##### Hemoglobin + Glucose Oxidase Nanoparticles (RBC-Coated)

In U87MG models, these nanoparticles generate reactive oxygen species (ROS), penetrate the BBB, and sustain fluorescent signaling within the tumor for 72 h, significantly inhibiting tumor growth [284].

##### Lipid–Amphiphilic Nanoformulations with PTX and PDL1-siRNA

In GL261 models, these exhibit selective cytotoxicity against GBM cells (50–70% in vitro). In vivo, median survival increased from 15 to 25 days, extending up to 45 days with the addition of PDL1-siRNA, without detectable toxicity [283].

##### Transferrin-Targeted Lipid Nanoparticles (Tf-PTX-LNPs)–Intranasal Administration

These nanoparticles facilitate transferrin receptor (TfR)-mediated BBB penetration, increasing brain drug concentration 5-fold in C_max_ and 4.9-fold in AUC_0–24_h. They induce strong cytotoxicity in U87MG cells, and in orthotopic models, they prolong survival with fewer side effects compared to systemic administration [284].

#### 4.1.2. In Vivo Studies

##### In Vivo Tests with PLGA-PEG Nanoparticles + antagomiRs

In a subcutaneous mouse model, nanoparticles exhibited an enhanced permeability and retention (EPR) effect, resulting in tumor volume reduction. The effect was stronger with cRGD-functionalized particles, allowing the use of lower doses of temozolomide (TMZ) [282].

##### Gold Nanoparticles (AuNCs, AuNTs) as Radiosensitizers

Folic acid-conjugated gold nanoclusters (FA-AuNCs) in C6 tumor models showed increased tumor accumulation. Combined with radiotherapy (6 Gy), median survival extended from 18 to 24.5 days [285]. Gold nanotriangles (AuNTs) in the U87MG model demonstrated no toxicity, favorable biodistribution, and enhanced radiotherapy efficacy after intravenous administration [285]. Other forms, including silver nanoparticles (AgNPs) and metal combinations administered intratumorally, increased apoptosis and autophagy without observable toxicity [285].

##### Doxorubicin-Loaded Nanoparticles Encapsulated in Exosomes (ENP_DOX)

These particles crossed the blood–brain barrier in the GL261 model, inducing immunogenic cell death (ICD), boosting immune response, increasing apoptosis, and improving survival in mice [286].

##### Fe_3_O_4_ Magnetic Nanoparticles with Antisense miR-10b (MN-Anti-miR10b)

Magnetic nanoparticles of approximately 25 nm were tracked via MRI and fluorescence, delivering therapeutic cargo to orthotopic tumors and demonstrating the feasibility of RNA interference therapy in vivo [285].

##### PLGA-Chlorotoxin (CTX) + Ionizing Radiation (IR)

PLGA nanoparticles conjugated with chlorotoxin, combined with radiotherapy, synergistically reduced MMP-2 expression and significantly decreased tumor volume in vivo [18,287].

##### CRLX101 (Camptothecin Conjugate)

In preclinical intravenous models, CRLX101 activated apoptosis and inhibited angiogenesis, reducing GBM growth in mouse xenografts [288].

#### 4.1.3. Summary of Efficacy and Safety

##### Efficacy

Nanoparticles enhance therapeutic accumulation in tumors via the EPR effect and ligand modification, enabling dose reduction of chemo-/radiotherapy. They promote apoptosis, inhibit angiogenesis, and modulate the tumor immune microenvironment, resulting in prolonged survival in murine models.

##### Safety

Most formulations show no significant organ toxicity. Selected materials (PLGA, gold, hemoglobin/glucose oxidase, lipid-based) demonstrated good tolerance with no hematologic or biochemical abnormalities. Potential accumulation of gold nanoparticles (e.g., in the liver) remains a concern.

Various strategies for BBB penetration are employed (ligands, surfactants, intranasal administration, focused ultrasound), many of which achieve efficient delivery of both cytotoxic and genetic therapeutic payloads.

### 4.2. Examples of Clinical Trials

Overview of current clinical trials applying nanotechnology in GBM treatment:

1. Gold Nanoparticles with RNAi: NU-0129 (Spherical Nucleic Acids)
  ○ Mechanism: Spherical gold-core nanoparticles coated with siRNA targeting the oncogene Bcl2L12.
  ○ Trial: Phase 0; eight patients with recurrent GBM received a very low intravenous dose of NU-0129 prior to tumor resection.
  ○ Results: Particles penetrated the tumor and reduced Bcl2L12 levels without significant toxicity (no grade 4/5 adverse events) [289].
  - Liposomes and Lipid-RNA Structures (LNPs): Vaccines and p53 Recycling
  - SGT-53: Liposomal pDNA encoding p53 with anti-TfR targeting, combined with temozolomide or radiotherapy. Phase II trial terminated early due to low recruitment (NCT02340156).
  - RNA–lipid NPs: RNA vaccine for newly diagnosed MGMT-unmethylated GBM patients (Phase I, NCT04573140) aimed at “reprogramming” the immune microenvironment [278].

2. Nanoparticles Enhancing Radiotherapy (Radiosensitizers)
  - AGuIX: Gadolinium-based nanoparticles administered intravenously alongside radiotherapy (30 Gy/10 fractions); Phase I confirmed safety [285].
  - Phase II formulations containing bismuth (AGuIX-Bi) are under development, showing improved radiotherapy efficacy and MR imaging in preclinical studies [290].

3. Photodynamic/Photothermal Therapy + Nanoparticles
  - Preclinical: Hybrid particles with angiopep and IR-780/mTHPC promoting PDT/PTT and selective apoptosis [291].
  - Other approach: Iridium(III) cores combined with gold nanoparticles, inducing devascularization and tumor elimination.

4. HDL-like Lipid Nanodiscs with LXR Agonists
  - Injection after tumor removal combined with radiotherapy in mouse models.
  - Results: >60% survival at 60 days, with immunological memory and rejection of subsequent tumors in 68% of mice. Preparations are underway for clinical trials.

5. Immunotherapeutic Approaches Using Nano-Elements
  - HSP-gp96: Peptide adjuvant in nanostructured vaccines (HSPPC), Phase I/II: immune response induced in 11/12 patients, progression delay in 41 individuals.
  - mRNA-LNP vaccines (similar to COVID-19 vaccines), currently in Phase I (NCT04573140) and further developed due to technological success [292].

Nanotechnology offers innovative, multimodal strategies—from RNAi, mRNA/LNP vaccines, to nanodiscs and radiosensitizers—with promising results in preclinical and early-phase clinical studies. However, only Phase II/III trials and standardized protocols (e.g., for SGT-53, AGuIX, RNA-LNP) will determine their true impact on patient survival and quality of life.

#### 4.2.1. Experimental Therapies Examples, Including Nanoparticles for Chemotherapeutic Drug Delivery

Experimental glioma therapies employ nanotechnology, biological mechanisms, and physical delivery methods to enhance the precision of drug delivery to the brain, reduce systemic side effects, stimulate antitumor immune responses, and integrate multiple modalities in a single system. Many of these strategies remain in preclinical or early clinical phases—larger clinical trials are needed to confirm their efficacy and safety. Below are selected experimental therapies with potential for future development.

1. Nanoparticles for Chemotherapy
  - Lipid-based Nanostructures—Temozolomide (TMZ):
    A comparative study of polymeric nanoparticles (PNP), solid lipid nanoparticles (SLN), and nanostructured lipid carriers (NLC) showed that TMZ-loaded NLCs (T-NLCs) exhibited superior anti-glioma efficacy—demonstrating better in vitro and in vivo outcomes, with stronger tumor growth inhibition and minimal side effects [293].
  - Surface-functionalized Liposomes:
    Liposomes loaded with TMZ, modified with anti-CD133 antibodies and angiopep-2, increased median survival from ~23 to ~49 days in mouse models. Co-loading TMZ with the BET inhibitor JQ1 and transferrin further enhanced therapeutic efficacy and reduced adverse effects [294].
  - Albumin and Metal Nanoparticles:
    Albumin nanoparticles (ABI-009) and polysiloxane gadolinium chelates (AGuIX) are being studied in combination with radiochemotherapy in ongoing phase I/II trials [291].
  - Platinum Conjugates and PEGylated Micelles:
    PEG-Glu micelles bearing cyclic RGD peptides facilitated oxaliplatin transport across the blood–brain barrier (BBB), significantly inhibiting tumor growth in animal models compared to standard drug formulations [295].
  - Chemodynamic Nanoreactors:
    DOX@MTP/HA-EGCG nanoparticles act as ‘cascade nanoreactors’ combining chemodynamic therapy (CDT) with chemotherapy. They efficiently cross the BBB, accumulate in tumors, and generate reactive oxygen species (ROS), resulting in enhanced cytotoxicity [296].
2. Technologies Supporting Drug Penetration
  - Focused Ultrasound (FUS)/Sonodynamic Therapy:
    FUS increases BBB permeability and, combined with microbubbles and agents such as doxorubicin or anti-PD-L1 antibodies, enhances drug accumulation in tumors. Sonodynamic therapy using fluorescent dyes and ultrasound showed clinical benefits, with documented neurological improvement in a patient case.
  - Convection-Enhanced Delivery (CED):
    Direct intracerebral delivery of drugs, such as radiolabeled liposomes (e.g., Re-186-containing 186RNL), improves dosing precision and reduces exposure of healthy tissue [297].
3. Gene Therapies and Biological Carriers
  - Liposomal Probes–p53 (SGT-53):
    Liposomal plasmid p53 combined with transferrin targeting. Despite promising preclinical data, the phase II trial was terminated due to low recruitment [3].
  - RNA/Lipid Nanoparticles (LNP-mRNA):
    mRNA carriers encoding, for example, CMV pp65, combined with dendritic cell immunotherapy, activated immune responses and are under phase I investigation (NCT04573140).
  - Oncolytic Viruses and Neural Stem Cell Carriers:
    Teserpaturev (Delytact, G47Δ)—an oncolytic HSV-1 virus approved in Japan after phase II, showing a ~84% one-year survival rate and median survival of ~20 months.
    NSC-CRAd-S-pk7—a replicating adenovirus (~70–90 nm) delivered via stem cells, shown to be safe in phase I.
    Toca 511 (vocimagene amiretrorepvec) + flucytosine: Viral gene therapy combining cytosine deaminase with a prodrug, in phase II/III for recurrent high-grade gliomas, with FDA/EMA priority designations.
4. Immunotherapy Supported by Nanotechnology
  - HSP-gp96 Biotherapeutic Vaccines:
    Protein complexes with tumor peptides in phase I/II trials showed activation of immune response, tumor immune infiltration, and a favorable safety profile [292].

#### 4.2.2. Conclusions and Future Research Directions

##### Personalized Targeted Therapy

Nanocarriers targeting specific receptors (TfR, LRP-1, CD44, EGFR) in brain and tumor tissues have demonstrated superior efficacy compared to conventional drugs in preclinical models. Combining therapies—chemotherapy, immunotherapy, photodynamic/photothermal therapy (PDT/PTT), radiotherapy, and gene therapy—in a single carrier system (e.g., lipid nanoparticles, nanoreactors) enables more effective tumor cell eradication.

##### Safe Brain Access

Minimally invasive, localized treatment modalities such as convection-enhanced delivery (CED), focused ultrasound (FUS), and sonodynamic therapy facilitate safer and more precise drug delivery to the brain.

##### Breakthroughs in Oncolysis and Immunoactivation

Oncolytic therapies (e.g., G47Δ) and immunotherapies (e.g., HSP-based vaccines, CAR-T cells) show promising results in early clinical phases.

### 4.3. Challenges in Clinical Application of Nanotechnology

Advantages of nanotechnology include facile functionalization, enhanced sensitivity, and tunable physicochemical properties. A key benefit in oncology is improved immune modulation: due to their small size, nanoparticles readily elicit immune responses upon administration. In cancer immunotherapy, nanoparticles serve as delivery vehicles for tumor antigens to antigen-presenting cells (APCs), potentiating immune activation [298]. Moreover, nanomaterials can deliver anticancer agents to both primary tumors and distant metastases [299]. However, potential adverse effects must be considered. Despite widespread use in oncology, concerns about nanoparticle-induced carcinogenicity persist. Exposure to nanomaterials has been linked to genetic aberrations in experimental studies [300]. Safety validation remains paramount, given that nanoparticles can enter the human body via inhalation and are internalized through endocytosis, potentially causing direct or indirect genetic damage. Such damage may disrupt normal cell cycle regulation, leading to genomic instability, mutations, or chromosomal abnormalities [301]. Ethical issues also arise in clinical trials involving nanomedicines. Participants must receive comprehensive and transparent information about experimental procedures. Concealment or incomplete disclosure during testing of new nanodrugs is unethical and violates the principle of informed consent [300].

## 5. Summary and Conclusions

### 5.1. Future of Nanotechnology in GBM Treatment

Nanoparticles of various architectures—liposomes, dendrimers, polymeric micelles—can be surface-modified (e.g., with monoclonal antibodies, peptides, transferrin) to enhance tumor targeting and reduce off-target distribution [89]. Examples include liposomal chemotherapy delivery systems (e.g., PEGylated liposomal doxorubicin) and temozolomide-loaded liposomes capable of crossing the blood–brain barrier. Combination therapies integrating immunotherapy and nanotechnology show significant promise. Nanoparticle-based theranostics (e.g., gold nano-membranes conjugated with 5-FU, anti-CD133, and anti-PD-L1 antibodies) can simultaneously exert chemotherapeutic, immunomodulatory, and photothermal effects. Nanocarriers also enable delivery of siRNA (e.g., NU-0129 gold-SNA platform) and p53 DNA (SGT-53) across the BBB. Integration with immunotherapeutic modalities—checkpoint inhibitors, vaccines, CAR-T cells, oncolytic viruses—represents a compelling research avenue [302]. To further advance and integrate nanotechnology with standard GBM treatments, priorities include the following: optimizing biocompatible nanoparticle design, precise targeting and controlled drug release, conducting phase trials of combination therapies, comprehensive safety and biodistribution assessments (nanotoxicology), long-term follow-up, and personalized medicine approaches through nanodiagnostics, biomarkers, and theranostics.

### 5.2. Conclusions

Nanoparticles can be designed to deliver chemotherapeutics, siRNA, or other drugs directly to GBM cells, bypassing healthy tissue and minimizing systemic toxicity. The BBB is a major obstacle in treating GBM. Nanoparticles, particularly those with specific surface modifications or using techniques like convection-enhanced delivery, can be engineered to cross this barrier and reach the tumor. Nanomaterials can be used as contrast agents for various imaging techniques like MRI, CT, and optical imaging, improving the accuracy and sensitivity of GBM detection and monitoring. Nanoparticles can be designed to carry multiple drugs simultaneously, enabling combination therapies to overcome drug resistance and improve treatment outcomes. Nanoparticles can be designed to target specific receptors, pathways, or even GBM stem cells within the tumor microenvironment. Nanoparticles can be designed to target specific receptors, pathways, or even GBM stem cells within the tumor microenvironment. Some nanoparticles can enhance the effectiveness of radiation therapy, making cancer cells more susceptible to radiation damage. Lipid-based nanoparticles that can encapsulate drugs and improve their stability and delivery. Made from biocompatible polymers, they can be designed to release drugs in a controlled manner. Superparamagnetic iron oxide nanoparticles (SPIONs) can be used for both imaging and drug delivery, leveraging their magnetic properties. Tailoring nanoparticle design and treatment strategies to individual patient characteristics and tumor profiles is a promising area for future research.

These novel therapies pose challenges and limitations. Long-term toxicity and stability data on nanomaterials remain scarce; their degradation and interactions with biological systems require further investigation. Tumor heterogeneity and uneven nanoparticle accumulation limit therapeutic efficacy and reproducibility. While many systems show promise preclinically, only a few have reached clinical trials (e.g., NU-0129, SGT-53, NanoTherm, PEG-Dox) [302]. Nanotechnology offers a multifaceted new perspective in combating GBM—improving drug delivery, enabling selective tumor destruction, enhancing radiotherapy, and providing a foundation for innovative immunotherapies. However, full clinical implementation necessitates robust safety studies, expanded clinical trials, and a deeper understanding of the tumor’s complex biology.

## Data Availability

No new data were created or analyzed in this study. Data sharing is not applicable to this article.
